# Methyl 3-(4-methoxy­phen­yl)-1-methyl-1,2,3,3a,4,11b-hexa­hydro­benzo[*f*]chromeno[4,3-*b*]pyrrole-3a-carboxyl­ate

**DOI:** 10.1107/S1600536810010056

**Published:** 2010-03-20

**Authors:** S. Thenmozhi, A. SubbiahPandi, S. Kathiravan, R. Raghunathan

**Affiliations:** a Department of Physics, Presidency College (Autonomous), Chennai 600 005, India; b Department of Organic Chemistry, University of Madras, Guindy Campus, Chennai 600 025, India

## Abstract

In the title compound, C_25_H_25_NO_4_, the pyrrolidine ring exhibits an envelope conformation and the tetra­hydro­pyran ring exhibits a half-chair conformation. The crystal structure is stabilized by inter­molecular C–H⋯π inter­actions.

## Related literature

For general background to the applications and biological activity of chromenopyrrole compounds, see: Caine (1993 ▶); Carlson (1993 ▶); Sokoloff *et al.* (1990 ▶); Wilner (1985 ▶); Biava *et al.* (2005 ▶); Fernandes *et al.* (2004 ▶); Borthwick *et al.* (2000 ▶); Jiang *et al.* (2004 ▶). For a related structure, see: Nirmala *et al.* (2009 ▶). For ring puckering analysis, see: Cremer & Pople (1975 ▶); Nardelli (1983 ▶).
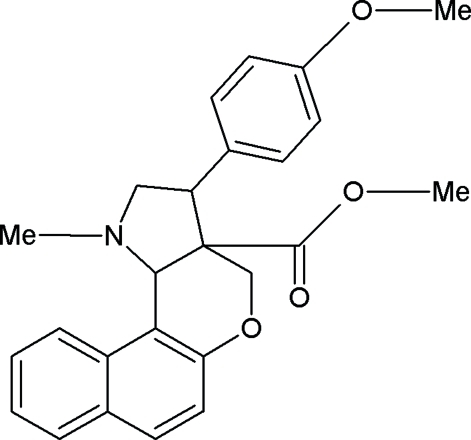


## Experimental

### 

#### Crystal data

C_25_H_25_NO_4_
*M* *_r_* = 403.46
Triclinic, 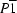

*a* = 7.9287 (5) Å
*b* = 10.8707 (6) Å
*c* = 11.6884 (7) Å
α = 95.662 (3)°
β = 92.332 (4)°
γ = 91.797 (4)°
*V* = 1001.05 (10) Å^3^
*Z* = 2
Mo *K*α radiation
μ = 0.09 mm^−1^
*T* = 293 K
0.25 × 0.22 × 0.19 mm

#### Data collection

Bruker APEXII CCD area-detector diffractometer
Absorption correction: multi-scan (*SADABS*; Sheldrick, 1996 ▶) *T*_min_ = 0.981, *T*_max_ = 0.985
21741 measured reflections
4776 independent reflections
3836 reflections with *I* > 2σ(*I*)
*R*_int_ = 0.050

#### Refinement

*R*[*F* ^2^ > 2σ(*F* ^2^)] = 0.050
*wR*(*F* ^2^) = 0.164
*S* = 1.05
4776 reflections
274 parameters
H-atom parameters constrained
Δρ_max_ = 0.31 e Å^−3^
Δρ_min_ = −0.34 e Å^−3^

### 

Data collection: *APEX2* (Bruker, 2004 ▶); cell refinement: *SAINT* (Bruker, 2004 ▶); data reduction: *SAINT*; program(s) used to solve structure: *SHELXS97* (Sheldrick, 2008 ▶); program(s) used to refine structure: *SHELXL97* (Sheldrick, 2008 ▶); molecular graphics: *ORTEP-3* (Farrugia, 1997 ▶); software used to prepare material for publication: *SHELXL97* and *PLATON* (Spek, 2009 ▶).

## Supplementary Material

Crystal structure: contains datablocks global, I. DOI: 10.1107/S1600536810010056/bt5203sup1.cif

Structure factors: contains datablocks I. DOI: 10.1107/S1600536810010056/bt5203Isup2.hkl

Additional supplementary materials:  crystallographic information; 3D view; checkCIF report

## Figures and Tables

**Table 1:** Hydrogen-bond geometry (Å, °) *Cg*4 is the centroid of the C1/C6–C10 ring.

*D*—H⋯*A*	*D*—H	H⋯*A*	*D*⋯*A*	*D*—H⋯*A*
C17—H17*B*⋯*Cg*4^i^	0.97	2.94	3.502 (2)	119

Symmetry code: (i) 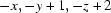.

**Fig. 1.**
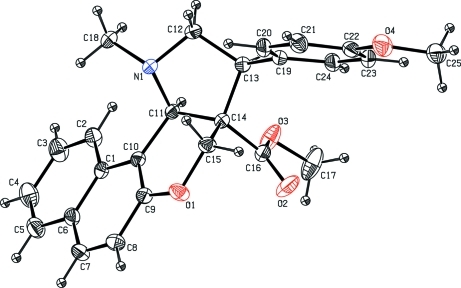
The structure of showing the atom-numbering scheme and intramolecular hydrogen bond. Displacement ellipsoids are drawn at the 30% probability level.

## supplementary crystallographic information

### Comment

Chromenopyrrole compounds are used in the treatment of impulsive disorders (Caine, 1993), parkinson's disease (Carlson, 1993), psychoses, memory disorders (Sokoloff *et al.*, 1990), anxiety and depression (Wilner, 1985). Pyrrole derivatives have good in vitro activities against mycobacteria and candidae (Biava *et al.*, 2005). These derivatives also possess anti-inflammatory (Fernandes *et al.*, 2004) and antiviral (Borthwick *et al.*, 2000) activities. It has also been shown that N-substituted pyrrole derivatives inhibit human immuno deficiency virus type-I (HIV-I) (Jiang *et al.*, 2004). Against this background, and in order to obtain detailed information on molecular conformation in the solid state, an X-ray study of the title compounds has been carried out.

The geometric parameters of the title molecule (Fig. 1) agree well with those reported for a similar structure (Nirmala *et al.*, 2009). The naphthalene ring system (C1—C10) and the benzene ring (C19—C24) are oriented at an angle of 71.1 (6)° with respect to each other. The pyrrolidine ring makes dihedral angles of 57.7 (7), 60.2 (7) and 64.0 (8)° with the naphthalene ring system and the tetrahydropyran and phenyl rings, respectively.

The pyrrolodine ring adopt an envelope conformation, with the puckering parameters q_2_ and φ (Cremer & Pople, 1975) and the smallest displacement asymmetric parameters,Δ, (Nardelli, 1983) as follows: q_2_=0.4285 (14) Å, φ=222.37 (19)°, Δ_s_(C13)= 3.46 (11) and the tetrahydropyran ring adopt a half-chair conformation, with the puckering parameters q_2_ and φ (Cremer & Pople, 1975) and the smallest displacement asymmetric parameters,Δ, (Nardelli, 1983) as follows: q_2_=0.3644 (14) Å, φ=73.5 (2)°, Δ_s_(C14)= 12.11 (13).

The crystal packing is stabilized by C–H···π (Table. 1) hydrogen bonds

### Experimental

A mixture of (2)-methyl-4-(1-formyl naphthalen-2-ylony)-3-(4-methoxy phenyl) but-2-enoate and sarcosine were refluxed in benzene for 20hr and the solvent was removed under reduced pressure. The crude product was subjected to column chromatography to get the pure product. Crystals were obtained by slow evaporation of a solution of the title compound in methanol at room temperature.

### Refinement

All H atoms were fixed geometrically and allowed to ride on their parent C atoms, with C—H distances fixed in the range 0.93–0.97 Å with U_iso_(H) = 1.5U_eq_(C) for methyl H and 1.2U_eq_(C) for other H atoms.

### Crystal data

**Table:**

C_25_H_25_NO_4_	*Z* = 2
*M**_r_* = 403.46	*F*(000) = 428
Triclinic, *P*1	*D*_x_ = 1.339 Mg m^−^^3^
Hall symbol: -P 1	Mo *K*α radiation, λ = 0.71073 Å
*a* = 7.9287 (5) Å	Cell parameters from 4776 reflections
*b* = 10.8707 (6) Å	θ = 1.8–28°
*c* = 11.6884 (7) Å	µ = 0.09 mm^−^^1^
α = 95.662 (3)°	*T* = 293 K
β = 92.332 (4)°	Block, colourless
γ = 91.797 (4)°	0.25 × 0.22 × 0.19 mm
*V* = 1001.05 (10) Å^3^	

### Data collection

**Table:**

Bruker APEXII CCD area-detector diffractometer	4776 independent reflections
Radiation source: fine-focus sealed tube	3836 reflections with *I* > 2σ(*I*)
graphite	*R*_int_ = 0.050
ω and φ scans	θ_max_ = 28.0°, θ_min_ = 1.8°
Absorption correction: multi-scan (*SADABS*; Sheldrick, 1996)	*h* = −10→10
*T*_min_ = 0.981, *T*_max_ = 0.985	*k* = −14→14
21741 measured reflections	*l* = −15→15

### Refinement

**Table:**

Refinement on *F*^2^	Primary atom site location: structure-invariant direct methods
Least-squares matrix: full	Secondary atom site location: difference Fourier map
*R*[*F*^2^ > 2σ(*F*^2^)] = 0.050	Hydrogen site location: inferred from neighbouring sites
*wR*(*F*^2^) = 0.164	H-atom parameters constrained
*S* = 1.05	*w* = 1/[σ^2^(*F*_o_^2^) + (0.099*P*)^2^ + 0.1502*P*] where *P* = (*F*_o_^2^ + 2*F*_c_^2^)/3
4776 reflections	(Δ/σ)_max_ < 0.001
274 parameters	Δρ_max_ = 0.31 e Å^−^^3^
0 restraints	Δρ_min_ = −0.34 e Å^−^^3^

### Special details

**Table:**

Geometry. All esds (except the esd in the dihedral angle between two l.s. planes) are estimated using the full covariance matrix. The cell esds are taken into account individually in the estimation of esds in distances, angles and torsion angles; correlations between esds in cell parameters are only used when they are defined by crystal symmetry. An approximate (isotropic) treatment of cell esds is used for estimating esds involving l.s. planes.
Refinement. Refinement of *F*^2^ against ALL reflections. The weighted *R*-factor wR and goodness of fit *S* are based on *F*^2^, conventional *R*-factors *R* are based on *F*, with *F* set to zero for negative *F*^2^. The threshold expression of *F*^2^ > σ(*F*^2^) is used only for calculating *R*-factors(gt) etc. and is not relevant to the choice of reflections for refinement. *R*-factors based on *F*^2^ are statistically about twice as large as those based on *F*, and *R*- factors based on ALL data will be even larger.

### Fractional atomic coordinates and isotropic or equivalent isotropic displacement parameters (Å^2^)

**Table:**

	*x*	*y*	*z*	*U*_iso_*/*U*_eq_	
C1	0.28588 (16)	0.64583 (12)	0.75782 (11)	0.0302 (3)	
C2	0.3939 (2)	0.54431 (14)	0.75627 (13)	0.0390 (3)	
H2	0.4605	0.5336	0.8216	0.047*	
C3	0.4023 (2)	0.46191 (16)	0.66078 (15)	0.0517 (4)	
H3	0.4730	0.3954	0.6628	0.062*	
C4	0.3065 (3)	0.47564 (17)	0.56016 (15)	0.0549 (5)	
H4	0.3152	0.4200	0.4951	0.066*	
C5	0.2008 (2)	0.57083 (16)	0.55850 (14)	0.0467 (4)	
H5	0.1366	0.5797	0.4917	0.056*	
C6	0.18576 (18)	0.65693 (13)	0.65573 (12)	0.0356 (3)	
C7	0.0693 (2)	0.75203 (15)	0.65447 (13)	0.0411 (4)	
H7	0.0030	0.7590	0.5881	0.049*	
C8	0.05266 (19)	0.83293 (15)	0.74783 (13)	0.0407 (3)	
H8	−0.0254	0.8948	0.7458	0.049*	
C9	0.15365 (17)	0.82395 (13)	0.84889 (12)	0.0322 (3)	
C10	0.27031 (16)	0.73371 (12)	0.85626 (11)	0.0281 (3)	
C11	0.36725 (15)	0.72609 (11)	0.96946 (11)	0.0269 (3)	
H11	0.3837	0.6393	0.9817	0.032*	
C12	0.57832 (17)	0.81208 (16)	1.10401 (12)	0.0403 (4)	
H12A	0.6351	0.8921	1.1238	0.048*	
H12B	0.6540	0.7484	1.1240	0.048*	
C13	0.41479 (16)	0.80366 (13)	1.16870 (11)	0.0311 (3)	
H13	0.4151	0.7246	1.2024	0.037*	
C14	0.27455 (15)	0.79025 (12)	1.07047 (11)	0.0280 (3)	
C15	0.22197 (17)	0.91646 (12)	1.03958 (12)	0.0331 (3)	
H15A	0.1609	0.9565	1.1023	0.040*	
H15B	0.3224	0.9673	1.0304	0.040*	
C16	0.12443 (17)	0.71790 (14)	1.10765 (12)	0.0349 (3)	
C17	0.0270 (3)	0.52672 (19)	1.1624 (2)	0.0776 (7)	
H17A	0.0029	0.5627	1.2381	0.116*	
H17B	0.0641	0.4440	1.1666	0.116*	
H17C	−0.0732	0.5248	1.1132	0.116*	
C18	0.66502 (17)	0.73843 (15)	0.91570 (13)	0.0394 (3)	
H18A	0.7666	0.7891	0.9293	0.059*	
H18B	0.6331	0.7310	0.8351	0.059*	
H18C	0.6842	0.6578	0.9396	0.059*	
C19	0.38660 (16)	0.90191 (13)	1.26574 (11)	0.0304 (3)	
C20	0.43808 (19)	1.02529 (14)	1.26490 (12)	0.0375 (3)	
H20	0.4962	1.0494	1.2028	0.045*	
C21	0.4050 (2)	1.11255 (14)	1.35383 (13)	0.0410 (3)	
H21	0.4418	1.1943	1.3516	0.049*	
C22	0.31722 (18)	1.07914 (14)	1.44666 (12)	0.0367 (3)	
C23	0.26739 (18)	0.95725 (15)	1.45086 (12)	0.0393 (3)	
H23	0.2102	0.9334	1.5135	0.047*	
C24	0.30326 (18)	0.87017 (14)	1.36077 (12)	0.0365 (3)	
H24	0.2701	0.7879	1.3645	0.044*	
C25	0.1930 (2)	1.1439 (2)	1.62332 (14)	0.0566 (5)	
H25A	0.0839	1.1100	1.5958	0.085*	
H25B	0.1796	1.2175	1.6741	0.085*	
H25C	0.2513	1.0843	1.6641	0.085*	
N1	0.53080 (13)	0.79497 (11)	0.98078 (9)	0.0307 (3)	
O1	0.11812 (13)	0.90921 (10)	0.93631 (9)	0.0414 (3)	
O2	−0.00673 (14)	0.76045 (13)	1.13242 (13)	0.0625 (4)	
O3	0.15812 (16)	0.59997 (11)	1.11635 (13)	0.0602 (4)	
O4	0.28770 (16)	1.17310 (11)	1.52900 (10)	0.0519 (3)	

### Atomic displacement parameters (Å^2^)

**Table:**

	*U*^11^	*U*^22^	*U*^33^	*U*^12^	*U*^13^	*U*^23^
C1	0.0296 (6)	0.0321 (6)	0.0298 (6)	−0.0027 (5)	0.0054 (5)	0.0073 (5)
C2	0.0454 (8)	0.0389 (7)	0.0336 (7)	0.0070 (6)	0.0048 (6)	0.0056 (6)
C3	0.0669 (11)	0.0452 (9)	0.0437 (9)	0.0145 (8)	0.0089 (8)	0.0019 (7)
C4	0.0749 (12)	0.0513 (10)	0.0367 (9)	0.0022 (9)	0.0062 (8)	−0.0065 (7)
C5	0.0546 (10)	0.0527 (9)	0.0317 (8)	−0.0054 (8)	−0.0013 (7)	0.0024 (6)
C6	0.0364 (7)	0.0404 (7)	0.0304 (7)	−0.0057 (6)	0.0025 (5)	0.0073 (6)
C7	0.0394 (8)	0.0520 (9)	0.0325 (7)	0.0001 (7)	−0.0067 (6)	0.0113 (6)
C8	0.0359 (7)	0.0474 (8)	0.0404 (8)	0.0099 (6)	−0.0026 (6)	0.0120 (6)
C9	0.0300 (6)	0.0353 (7)	0.0320 (7)	0.0030 (5)	0.0010 (5)	0.0068 (5)
C10	0.0252 (6)	0.0313 (6)	0.0288 (6)	0.0005 (5)	0.0028 (5)	0.0072 (5)
C11	0.0239 (6)	0.0301 (6)	0.0279 (6)	0.0043 (5)	0.0040 (5)	0.0070 (5)
C12	0.0250 (6)	0.0628 (10)	0.0322 (7)	0.0038 (6)	0.0005 (5)	0.0003 (6)
C13	0.0277 (6)	0.0376 (7)	0.0288 (6)	0.0042 (5)	0.0018 (5)	0.0061 (5)
C14	0.0239 (6)	0.0327 (6)	0.0280 (6)	0.0034 (5)	0.0032 (5)	0.0034 (5)
C15	0.0308 (6)	0.0344 (7)	0.0339 (7)	0.0069 (5)	−0.0009 (5)	0.0008 (5)
C16	0.0291 (7)	0.0443 (8)	0.0309 (7)	−0.0018 (6)	0.0049 (5)	0.0012 (6)
C17	0.0787 (15)	0.0543 (11)	0.1033 (18)	−0.0161 (10)	0.0519 (13)	0.0120 (11)
C18	0.0269 (7)	0.0548 (9)	0.0378 (7)	0.0067 (6)	0.0085 (6)	0.0064 (6)
C19	0.0251 (6)	0.0402 (7)	0.0262 (6)	0.0019 (5)	0.0001 (5)	0.0052 (5)
C20	0.0391 (7)	0.0441 (8)	0.0302 (7)	−0.0039 (6)	0.0053 (6)	0.0085 (6)
C21	0.0476 (8)	0.0380 (7)	0.0371 (8)	−0.0034 (6)	−0.0010 (6)	0.0052 (6)
C22	0.0341 (7)	0.0460 (8)	0.0287 (7)	0.0051 (6)	−0.0044 (5)	−0.0010 (6)
C23	0.0360 (7)	0.0540 (9)	0.0280 (7)	−0.0030 (6)	0.0058 (6)	0.0050 (6)
C24	0.0384 (7)	0.0408 (7)	0.0305 (7)	−0.0043 (6)	0.0024 (6)	0.0066 (6)
C25	0.0484 (9)	0.0837 (13)	0.0347 (8)	0.0121 (9)	0.0016 (7)	−0.0109 (8)
N1	0.0221 (5)	0.0412 (6)	0.0296 (6)	0.0029 (4)	0.0037 (4)	0.0051 (4)
O1	0.0434 (6)	0.0443 (6)	0.0364 (6)	0.0202 (5)	−0.0060 (4)	0.0007 (4)
O2	0.0303 (6)	0.0720 (9)	0.0899 (10)	0.0074 (5)	0.0210 (6)	0.0215 (7)
O3	0.0556 (7)	0.0393 (6)	0.0896 (10)	−0.0023 (5)	0.0395 (7)	0.0125 (6)
O4	0.0600 (7)	0.0559 (7)	0.0375 (6)	0.0077 (6)	0.0027 (5)	−0.0085 (5)

### Geometric parameters (Å, °)

**Table:**

C1—C2	1.4171 (19)	C14—C15	1.5193 (18)
C1—C6	1.4234 (19)	C15—O1	1.4274 (16)
C1—C10	1.4331 (18)	C15—H15A	0.9700
C2—C3	1.366 (2)	C15—H15B	0.9700
C2—H2	0.9300	C16—O2	1.1864 (17)
C3—C4	1.397 (3)	C16—O3	1.3304 (19)
C3—H3	0.9300	C17—O3	1.444 (2)
C4—C5	1.353 (3)	C17—H17A	0.9600
C4—H4	0.9300	C17—H17B	0.9600
C5—C6	1.411 (2)	C17—H17C	0.9600
C5—H5	0.9300	C18—N1	1.4503 (16)
C6—C7	1.408 (2)	C18—H18A	0.9600
C7—C8	1.346 (2)	C18—H18B	0.9600
C7—H7	0.9300	C18—H18C	0.9600
C8—C9	1.413 (2)	C19—C24	1.3823 (19)
C8—H8	0.9300	C19—C20	1.391 (2)
C9—O1	1.3547 (17)	C20—C21	1.376 (2)
C9—C10	1.3757 (18)	C20—H20	0.9300
C10—C11	1.5137 (17)	C21—C22	1.385 (2)
C11—N1	1.4715 (17)	C21—H21	0.9300
C11—C14	1.5365 (17)	C22—O4	1.3652 (17)
C11—H11	0.9800	C22—C23	1.377 (2)
C12—N1	1.4661 (18)	C23—C24	1.389 (2)
C12—C13	1.5321 (19)	C23—H23	0.9300
C12—H12A	0.9700	C24—H24	0.9300
C12—H12B	0.9700	C25—O4	1.415 (2)
C13—C19	1.5078 (18)	C25—H25A	0.9600
C13—C14	1.5584 (18)	C25—H25B	0.9600
C13—H13	0.9800	C25—H25C	0.9600
C14—C16	1.5083 (19)		
			
C2—C1—C6	116.85 (13)	C11—C14—C13	102.15 (9)
C2—C1—C10	123.60 (12)	O1—C15—C14	112.60 (11)
C6—C1—C10	119.53 (12)	O1—C15—H15A	109.1
C3—C2—C1	121.38 (14)	C14—C15—H15A	109.1
C3—C2—H2	119.3	O1—C15—H15B	109.1
C1—C2—H2	119.3	C14—C15—H15B	109.1
C2—C3—C4	121.19 (16)	H15A—C15—H15B	107.8
C2—C3—H3	119.4	O2—C16—O3	122.75 (14)
C4—C3—H3	119.4	O2—C16—C14	125.01 (14)
C5—C4—C3	119.19 (15)	O3—C16—C14	112.14 (11)
C5—C4—H4	120.4	O3—C17—H17A	109.5
C3—C4—H4	120.4	O3—C17—H17B	109.5
C4—C5—C6	121.58 (15)	H17A—C17—H17B	109.5
C4—C5—H5	119.2	O3—C17—H17C	109.5
C6—C5—H5	119.2	H17A—C17—H17C	109.5
C7—C6—C5	120.90 (14)	H17B—C17—H17C	109.5
C7—C6—C1	119.31 (13)	N1—C18—H18A	109.5
C5—C6—C1	119.77 (14)	N1—C18—H18B	109.5
C8—C7—C6	120.99 (13)	H18A—C18—H18B	109.5
C8—C7—H7	119.5	N1—C18—H18C	109.5
C6—C7—H7	119.5	H18A—C18—H18C	109.5
C7—C8—C9	120.09 (13)	H18B—C18—H18C	109.5
C7—C8—H8	120.0	C24—C19—C20	117.15 (13)
C9—C8—H8	120.0	C24—C19—C13	119.33 (12)
O1—C9—C10	124.81 (12)	C20—C19—C13	123.51 (12)
O1—C9—C8	113.03 (12)	C21—C20—C19	121.51 (13)
C10—C9—C8	122.12 (13)	C21—C20—H20	119.2
C9—C10—C1	117.93 (12)	C19—C20—H20	119.2
C9—C10—C11	118.97 (12)	C20—C21—C22	120.28 (14)
C1—C10—C11	122.96 (11)	C20—C21—H21	119.9
N1—C11—C10	114.52 (10)	C22—C21—H21	119.9
N1—C11—C14	101.23 (10)	O4—C22—C23	124.85 (14)
C10—C11—C14	111.22 (10)	O4—C22—C21	115.66 (14)
N1—C11—H11	109.9	C23—C22—C21	119.49 (13)
C10—C11—H11	109.9	C22—C23—C24	119.42 (13)
C14—C11—H11	109.9	C22—C23—H23	120.3
N1—C12—C13	106.97 (11)	C24—C23—H23	120.3
N1—C12—H12A	110.3	C19—C24—C23	122.11 (14)
C13—C12—H12A	110.3	C19—C24—H24	118.9
N1—C12—H12B	110.3	C23—C24—H24	118.9
C13—C12—H12B	110.3	O4—C25—H25A	109.5
H12A—C12—H12B	108.6	O4—C25—H25B	109.5
C19—C13—C12	118.15 (12)	H25A—C25—H25B	109.5
C19—C13—C14	115.05 (10)	O4—C25—H25C	109.5
C12—C13—C14	103.36 (10)	H25A—C25—H25C	109.5
C19—C13—H13	106.5	H25B—C25—H25C	109.5
C12—C13—H13	106.5	C18—N1—C12	110.87 (11)
C14—C13—H13	106.5	C18—N1—C11	115.47 (11)
C16—C14—C15	110.32 (11)	C12—N1—C11	106.57 (10)
C16—C14—C11	114.99 (11)	C9—O1—C15	118.16 (10)
C15—C14—C11	108.79 (10)	C16—O3—C17	115.85 (14)
C16—C14—C13	109.58 (11)	C22—O4—C25	117.54 (14)
C15—C14—C13	110.74 (11)		
			
C6—C1—C2—C3	0.6 (2)	C12—C13—C14—C15	−87.13 (13)
C10—C1—C2—C3	178.95 (14)	C19—C13—C14—C11	158.84 (11)
C1—C2—C3—C4	1.0 (3)	C12—C13—C14—C11	28.58 (13)
C2—C3—C4—C5	−1.5 (3)	C16—C14—C15—O1	−69.67 (14)
C3—C4—C5—C6	0.4 (3)	C11—C14—C15—O1	57.34 (14)
C4—C5—C6—C7	−176.93 (16)	C13—C14—C15—O1	168.84 (10)
C4—C5—C6—C1	1.3 (2)	C15—C14—C16—O2	−14.5 (2)
C2—C1—C6—C7	176.53 (13)	C11—C14—C16—O2	−137.97 (16)
C10—C1—C6—C7	−1.9 (2)	C13—C14—C16—O2	107.68 (17)
C2—C1—C6—C5	−1.7 (2)	C15—C14—C16—O3	169.18 (12)
C10—C1—C6—C5	179.87 (13)	C11—C14—C16—O3	45.69 (16)
C5—C6—C7—C8	178.81 (15)	C13—C14—C16—O3	−68.65 (15)
C1—C6—C7—C8	0.6 (2)	C12—C13—C19—C24	−145.25 (14)
C6—C7—C8—C9	0.5 (2)	C14—C13—C19—C24	92.10 (15)
C7—C8—C9—O1	−177.94 (14)	C12—C13—C19—C20	35.71 (18)
C7—C8—C9—C10	−0.3 (2)	C14—C13—C19—C20	−86.94 (16)
O1—C9—C10—C1	176.35 (12)	C24—C19—C20—C21	−1.1 (2)
C8—C9—C10—C1	−1.0 (2)	C13—C19—C20—C21	177.95 (13)
O1—C9—C10—C11	0.5 (2)	C19—C20—C21—C22	−0.7 (2)
C8—C9—C10—C11	−176.85 (12)	C20—C21—C22—O4	−178.57 (13)
C2—C1—C10—C9	−176.25 (13)	C20—C21—C22—C23	1.8 (2)
C6—C1—C10—C9	2.07 (19)	O4—C22—C23—C24	179.22 (13)
C2—C1—C10—C11	−0.6 (2)	C21—C22—C23—C24	−1.2 (2)
C6—C1—C10—C11	177.76 (11)	C20—C19—C24—C23	1.7 (2)
C9—C10—C11—N1	−93.00 (14)	C13—C19—C24—C23	−177.37 (12)
C1—C10—C11—N1	91.35 (14)	C22—C23—C24—C19	−0.6 (2)
C9—C10—C11—C14	21.00 (17)	C13—C12—N1—C18	−150.22 (12)
C1—C10—C11—C14	−154.65 (12)	C13—C12—N1—C11	−23.81 (14)
N1—C12—C13—C19	−132.33 (12)	C10—C11—N1—C18	−74.85 (14)
N1—C12—C13—C14	−3.96 (15)	C14—C11—N1—C18	165.40 (11)
N1—C11—C14—C16	−161.46 (11)	C10—C11—N1—C12	161.56 (11)
C10—C11—C14—C16	76.47 (14)	C14—C11—N1—C12	41.81 (12)
N1—C11—C14—C15	74.25 (12)	C10—C9—O1—C15	7.9 (2)
C10—C11—C14—C15	−47.82 (14)	C8—C9—O1—C15	−174.55 (12)
N1—C11—C14—C13	−42.87 (12)	C14—C15—O1—C9	−37.70 (17)
C10—C11—C14—C13	−164.94 (10)	O2—C16—O3—C17	−2.3 (3)
C19—C13—C14—C16	−78.79 (14)	C14—C16—O3—C17	174.09 (16)
C12—C13—C14—C16	150.94 (11)	C23—C22—O4—C25	−2.7 (2)
C19—C13—C14—C15	43.13 (15)	C21—C22—O4—C25	177.75 (14)

### Hydrogen-bond geometry (Å, °)

**Table:**

Cg4 is the centroid of the C1/C6–C10 ring.

**Table:**

*D*—H···*A*	*D*—H	H···*A*	*D*···*A*	*D*—H···*A*
C11—H11···O3	0.98	2.48	2.8514 (18)	102
C13—H13···O3	0.98	2.53	2.9610 (18)	107
C17—H17B···Cg4^i^	0.97	2.94	3.502 (2)	119

Symmetry codes: (i) −*x*, −*y*+1, −*z*+2.
